# Supplementary material for: Learning curve and surgical outcome of robotic assisted colorectal surgery with ERAS program
Source: Sci Rep. 2022 Nov 29;12:20566. doi: 10.1038/s41598-022-24665-w (PMC9709162; doi:10.1038/s41598-022-24665-w)
Supplement: Supplementary file 2 — Supplementary Information 2. [file 41598_2022_24665_MOESM2_ESM.zip › RAL Raw data-Ñ[▒K/053/OP_ñw╝╨░O▒Kñσ.pdf]

正式摘要(電子簽章：000000208398589)

W52 / W52

臺中榮民總醫院

Operation Note

[redacted]

- 手術日期：2021/01/18
- 手術醫師：VS 蔣鋒帆，CR 林陳彥，CR 陳映菁，R 陳凱祥
- Anesthesia：GE
- Position：Lithotomy
- Aseptic agents：Betadine Aqua，Zephnan

Diagnosis

- Pre-operative diagnosis : MUCOAL MELANOMA OF RECTUM
- Post-operative diagnosis : Rectum melanoma s/p CCRT s/p robotic assisted APR

Surgical Indication

- For removing the primary tumor as possible

Operation

- 1. Robotic (XI) assisted abdominal-perineal resection with end S-colostomy
  2. Intraoperative colonoscopy : (-).
  3. High ligation (+) : At IMA root. Left colic artery and IMV were also ligated via robotic endoclips.
  4. Take down from the splenic flexure : (-)
  5. Robotic endoGIA (45-4.3) X1 was applied to resected the S-colon.
  6. Proximal cut end: 10 cm
  7. Two 12 Fr. penrose drainage tube were placed from the perineal wound into the pelvic cavity.
  8. The perineal wound was closed with interrupted 2-0 Nylon and then covered with gauze.

Finding

- 1. The tumor may be locally advanced. It was severely attached to the nearby structure, especially the posterior wall of vagina. (May be post CCRT related ?). An ovary like tissue was also severely attached to the rectum surface. It was later resected for sampling, marked as "Peritonuem"
  2. Several mass lesions over uterus, favored uterine myoma.
  3. Suspicious lymphnode over the para-aortic area was resected and it was also send for sampling.
  4. No obvious ascites was found.

Procedure

- 1. General endotracheal anesthesia was induced and then the patient was placed in modified Trendelenburg lithotomy position.
  2. Preoperative antibiotics were given. A Foley catheter and nasogastric tube were placed.
  3. The abdomen was prepped and draped in standard sterile fashion.
  4. A transverse incision was made above the umbilicus and a 8 mm trocar was inserted into the peritoneal cavity by open method. Peumoperitoneum was established with CO2 inflation.
  5. Laparoscopy was inserted and then another 8mm trocar was inserted over LUQ area of abdomen. A 12 mm trochar over RLQ area of abdomen. Another 8mm trochar over RLQ of abdomen. Finally, a 5 mm trocar was inserted over RUQ area as an assist-port. The operative findings were mentioned as above.
  6. The sigmoid colon was held by grasper and the peritoneum overlying the mesentery was scored

- with electrocautery.
  7. The inferior mesentery artery was identified, ligated above the left colic artery with robotic endoclips, and transected.
  8. Dissection was done above the Gerota's fascia and toward the peritoneal attachments.
  9. The colon was mobilized from retroperitoneum overlying the sacral promontory and into the pelvis.

  10. The incision was extended laterally and medially across the peritoneal reflection. The adhesion was dissected as possible.
  11. The rectum was severely attached to the posterior wall of vagina. Dissection was performed carefully, until the vagina was freely movable from the rectum.
  12. The mesorectum was then mobilized using sharp dissection over the level of pelvic floor muscle. Then distal descending colon and rectum were mobilized as much as possible. The rectum was carefully elevated until the levator sling was identified.
  13. The desired S-colon part was then resected via firing one staple of robotic EndoGIA (45-4.3).
  14. Then we shifted to perineum approach. (Robotic arms were no longer needed.)
  15. An elliptical incision was made encompassing the anus and extending up. Dissection then progressed through subcutaneous tissues circumferentially.
  16. The abdomen was entered posterior to the anus in the midline. The levators were then divided with electrocautery beginning posteriorly and progressing anteriorly.
  17. The specimen was delivered through this posterior incision into the perineal field. The remaining attahced part was further dissected thoroughly with electrical cautery.
  18. The specimen (rectum) was then taken out from the perineal wound. Hemostasis was achieved in the pelvis and perineal wound area.
  19. The anal canal was irrigated with distil water thoroughly. Hemostasis was done carefully.
  20. Two 12 Fr. penrose drains were placed in the pelvis and brought out through the wound in the perineum.
  21. The perineum wound was closed with interrupted method via 2-O Nylon.
  22. We shifted our procedure back to end S-colon stomy formation. The desired ostomy site was made over LLQ of the abdomen.
  23. An oval incision was made and the incision was deepended through subcutaneous tissues, muscular and fascial layers until an opening sufficient for at least two fingers was created.
  24. The end of S-colon was then delivered through this opening without tension or torsion. It was fixed to the peritoneum medially with interrupted 2-0 silk and laterally to the peritoneum in such a manner as to eliminate the lateral trap.
  25. The trochar fascia wounds were all closed with interrupted sutures via 1-O Vicryl.
  26. Massive N/S was applied to irrigate the wound.
  27. The wound was further closed with 3-O Vicryl, and the skin was applied with SurgiSeal. Finally covered with Medipore.
  28. The colostomy was then mature with multiple interrupted sutures via 4-O Monocryl.
  29. The patient tolerated the procedure well and was brought to the POR under relative stable situation.
- Estimated blood loss: 300 c.c
- 器械數量: 正確
- 紗布數量: 正確

Image

- PAINT
  - abdomen

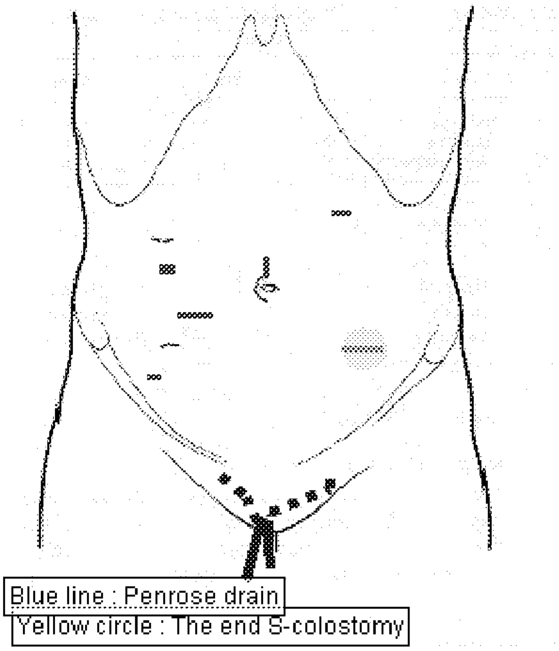

- colon & rectum

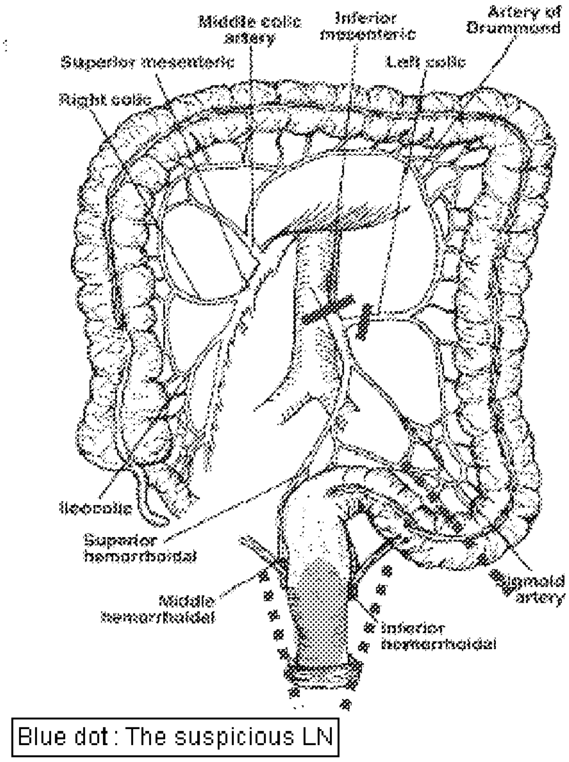

- The resected rectum

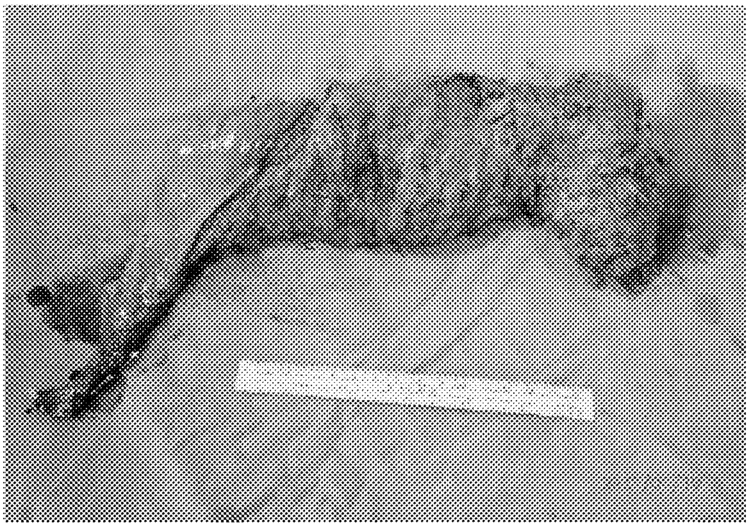

The suspicious tissue, may be ovary, was severely attached to the rectum surface (It was resected for pathology sampling, marked as " Peritoneum")

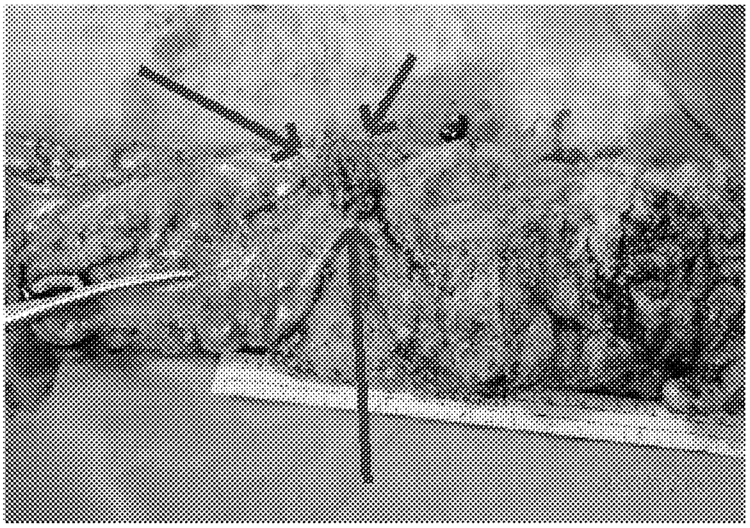

The appearance of the tumor

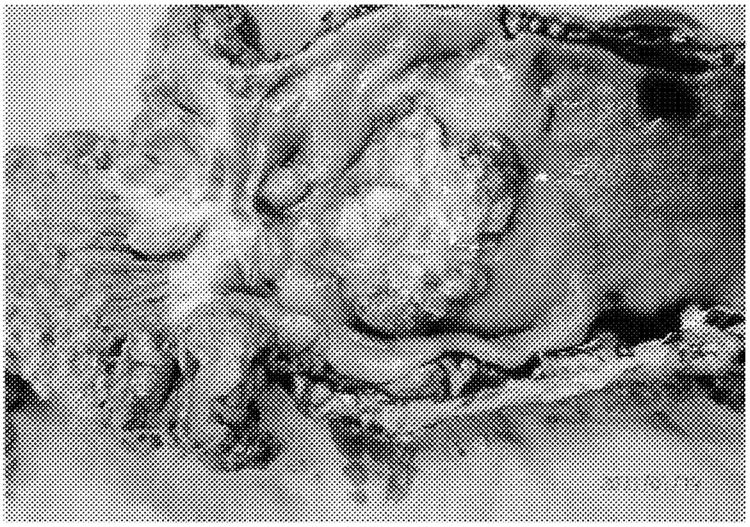

主任 2062F蔣鋒帆 / 醫師 2301H林陳彥

正式摘要**(**電子簽章：**000000208398589)**

W52 / W52

doctorRank:B
